# Advancements in CRISPR screens for the development of cancer immunotherapy strategies

**DOI:** 10.1016/j.omto.2023.100733

**Published:** 2023-10-05

**Authors:** Yan-Ruide Li, Zibai Lyu, Yanxin Tian, Ying Fang, Yichen Zhu, Yuning Chen, Lili Yang

**Affiliations:** 1 Department of Microbiology, Immunology & Molecular Genetics, University of California, Los Angeles, Los Angeles, CA 90095, USA; 2 Eli and Edythe Broad Center of Regenerative Medicine and Stem Cell Research, University of California, Los Angeles, Los Angeles, CA 90095, USA; 3 Jonsson Comprehensive Cancer Center, David Geffen School of Medicine, University of California, Los Angeles, Los Angeles, CA 90095, USA; 4 Molecular Biology Institute, University of California, Los Angeles, Los Angeles, CA 90095, USA

**Keywords:** clustered regularly interspaced short palindromic repeats (CRISPR) screen, cancer immunotherapy, T cells, NK cells, chimeric antigen receptor (CAR)-engineered T (CAR-T) cells, genetic engineering, gene discovery, tumor immunology

## Abstract

CRISPR screen technology enables systematic and scalable interrogation of gene function by using the CRISPR-Cas9 system to perturb gene expression. In the field of cancer immunotherapy, this technology has empowered the discovery of genes, biomarkers, and pathways that regulate tumor development and progression, immune reactivity, and the effectiveness of immunotherapeutic interventions. By conducting large-scale genetic screens, researchers have successfully identified novel targets to impede tumor growth, enhance anti-tumor immune responses, and surmount immunosuppression within the tumor microenvironment (TME). Here, we present an overview of CRISPR screens conducted in tumor cells for the purpose of identifying novel therapeutic targets. We also explore the application of CRISPR screens in immune cells to propel the advancement of cell-based therapies, encompassing T cells, natural killer cells, dendritic cells, and macrophages. Furthermore, we outline the crucial components necessary for the successful implementation of immune-specific CRISPR screens and explore potential directions for future research.

## Graphical abstract

**Figure:**
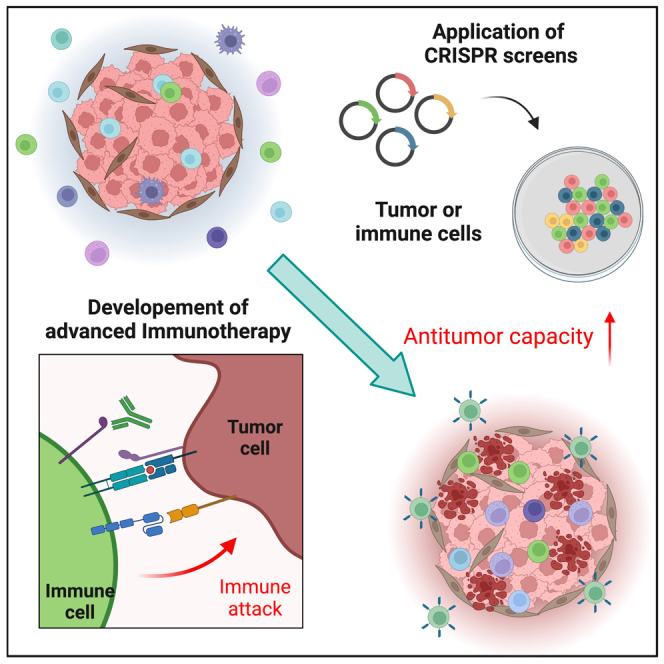

## Teaser

Li and colleagues present a review of CRISPR screen applications in the development of cancer immunotherapy, summarizing the use of CRISPR screens in tumor cells to identify novel therapeutic targets and in immune cells to enhance their therapeutic potential.

## Introduction

The discovery of CRISPR-Cas9 as a versatile genome editing tool paved the way for its application in functional genomics.^1^^,^^2^^,^^3^^,^^4^^,^^5^^,^^6^^,^^7^^,^^8^^,^^9^^,^^10^^,^^11^ Initially recognized for its gene-editing capabilities, scientists soon realized the immense potential of CRISPR-Cas9 beyond mere editing and began exploring its applications in large-scale screening experiments.^12^^,^^13^^,^^14^^,^^15^^,^^16^^,^^17^ This led to the development of CRISPR screen technologies, which enable systematic manipulation of gene function and the identification of genes associated with specific phenotypes or biological processes.^18^^,^^19^^,^^20^^,^^21^

CRISPR screen technologies involve the use of guide RNAs (gRNAs) to guide the Cas9 enzyme to precise genomic locations, where it induces double-stranded breaks.^1^^,^^2^^,^^22^ These breaks stimulate DNA repair mechanisms, resulting in either random insertions or deletions that disrupt gene function or the replacement of the target gene with an exogenous DNA sequence.^1^^,^^2^ The resulting perturbation in gene expression allows researchers to assess the functional consequences of individual gene knockouts (KOs) or knockins in a high-throughput manner, and this can be accomplished through two primary approaches: pooled CRISPR screens and multiplexed arrayed CRISPR screens.^12^

Prior to the advent of CRISPR-based screens, loss-of-function screens relied primarily on RNAi-based technologies. RNAi uses small interfering RNA and short hairpin RNA to bind to messenger RNA of the target gene, thereby controlling its protein translation.^23^ Because of the intrinsic difference between gene KO and knockdown, CRISPR screens can produce more consistent genotypes than the hypomorphic mutations generated by RNAi screens. Parallel comparative studies have shown that CRISPR screens identified essential genes with greater accuracy and mitigated off-target effects more effectively than RNAi screens.^24^ As a pre-transcriptional regulatory tool, CRISPR-based technologies are capable to screen noncoding regions, offering a broader coverage compared with RNAi screens.

The CRISPR screen workflow encompasses several essential steps: gRNA design for targeting specific genes, construction of a gRNA library, delivery of CRISPR components via viral vectors or transfection, phenotypic or functional selection to identify desired genetic alterations, subsequent sequencing and data analysis, and validation through additional experiments.^12^^,^^15^ This comprehensive approach enables various applications, such as the discovery of gene functions, identification of potential drug targets, functional annotation of genomes, and mapping of pathways and disease mechanisms.^25^^,^^26^^,^^27^ Notably, the CRISPR screen surpasses previous screening methods because of its ability to investigate gene function at a genome-wide scale, facilitating the identification of both known and novel genes involved in specific biological processes or disease phenotypes.^12^^,^^16^ Furthermore, CRISPR screen exhibits versatility by accommodating different cell types, including primary cells and organoids, thereby broadening its utility across diverse research domains.^28^^,^^29^^,^^30^

In the field of cancer immunotherapy, CRISPR screens have facilitated the discovery of genes involved in immune cell activation, immune checkpoint regulation, and antigen presentation.^31^^,^^32^^,^^33^^,^^34^^,^^35^^,^^36^ These discoveries have led to the development of innovative immunomodulatory drugs and optimized therapies. Additionally, CRISPR screens have revealed the mechanisms behind immunotherapy resistance, identifying genes involved in immune evasion and tumor immune escape.^37^^,^^38^^,^^39^ This knowledge has guided the design of combination therapies targeting multiple resistance mechanisms to overcome treatment resistance and improve patient outcomes. Moreover, CRISPR screens have expedited personalized cancer immunotherapies by identifying genetic markers associated with treatment response.^40^^,^^41^^,^^42^ This enables tailored treatment strategies based on individual genetic profiles, maximizing efficacy and minimizing side effects.

**Figure 1:**
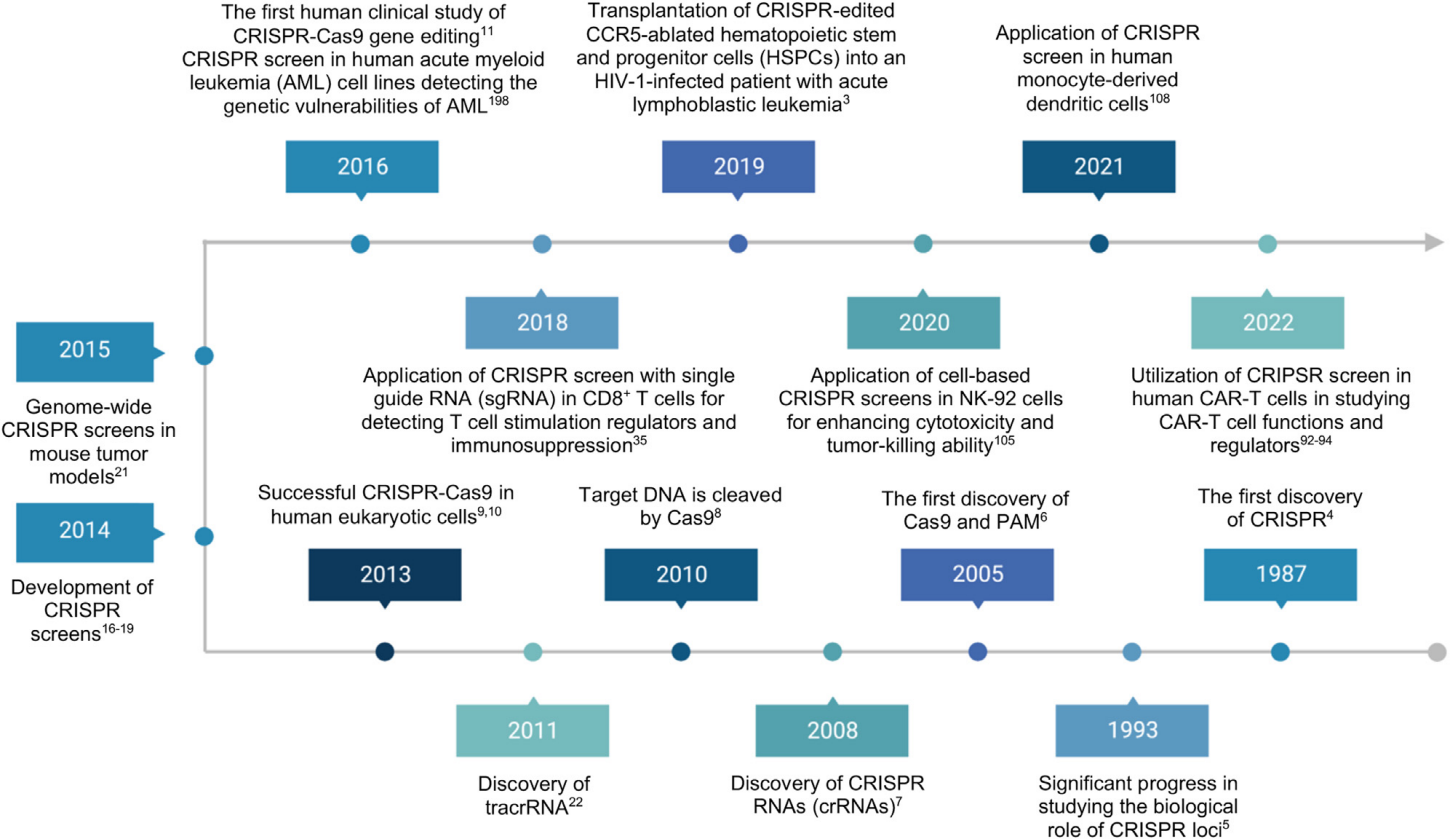
The chronological progression of CRISPR screens in cancer immunotherapy research

**Figure 2:**
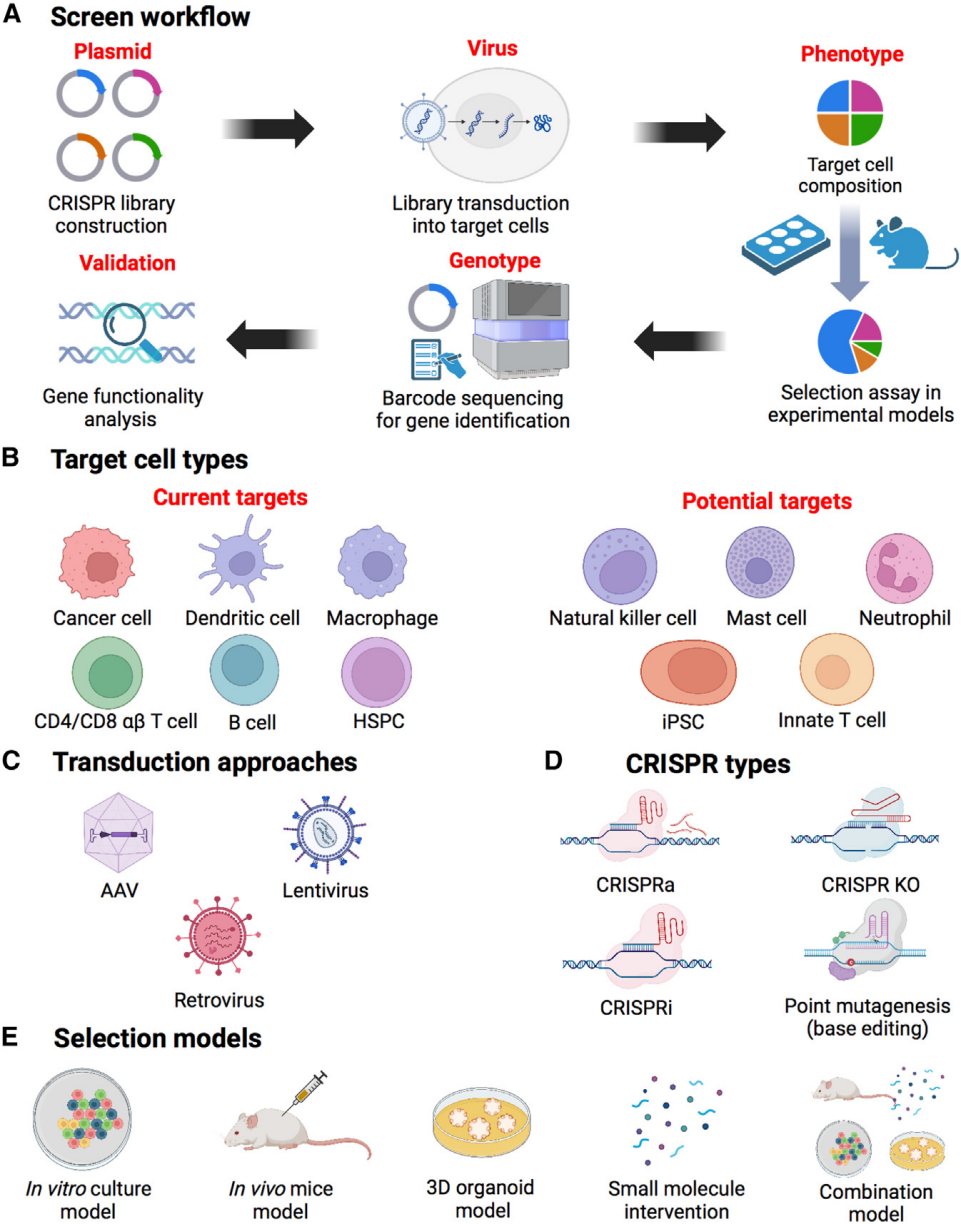
CRISPR screen overview (A) Schematic showing the CRISPR screen workflow. A genome-wide CRISPR library was constructed and introduced into the target cells via transduction. Various selection assays, including *in vitro*, *in vivo*, or other methods, were used to manipulate the cellular composition. Subsequently, the identification of specific genes of interest was achieved through the isolation of genomic DNA and barcode sequencing. Further experimental validation was conducted to investigate and characterize the functional role of the identified genes. (B) Current or potential cell types that could be targeted by CRISPR screen. (C) Three transduction approaches used in CRISPR screen. (D) Four major types of CRISPR gene editing strategies. (E) Selection models involved in the CRISPR screen procedure. HSPC, hematopoietic stem and progenitor cell; iPSC, induced pluripotent stem cell; AAV, adeno-associated virus; CRISPRa, CRISPR activation; CRISPRi, CRISPR interference; KO, knockout.

**Figure 3:**
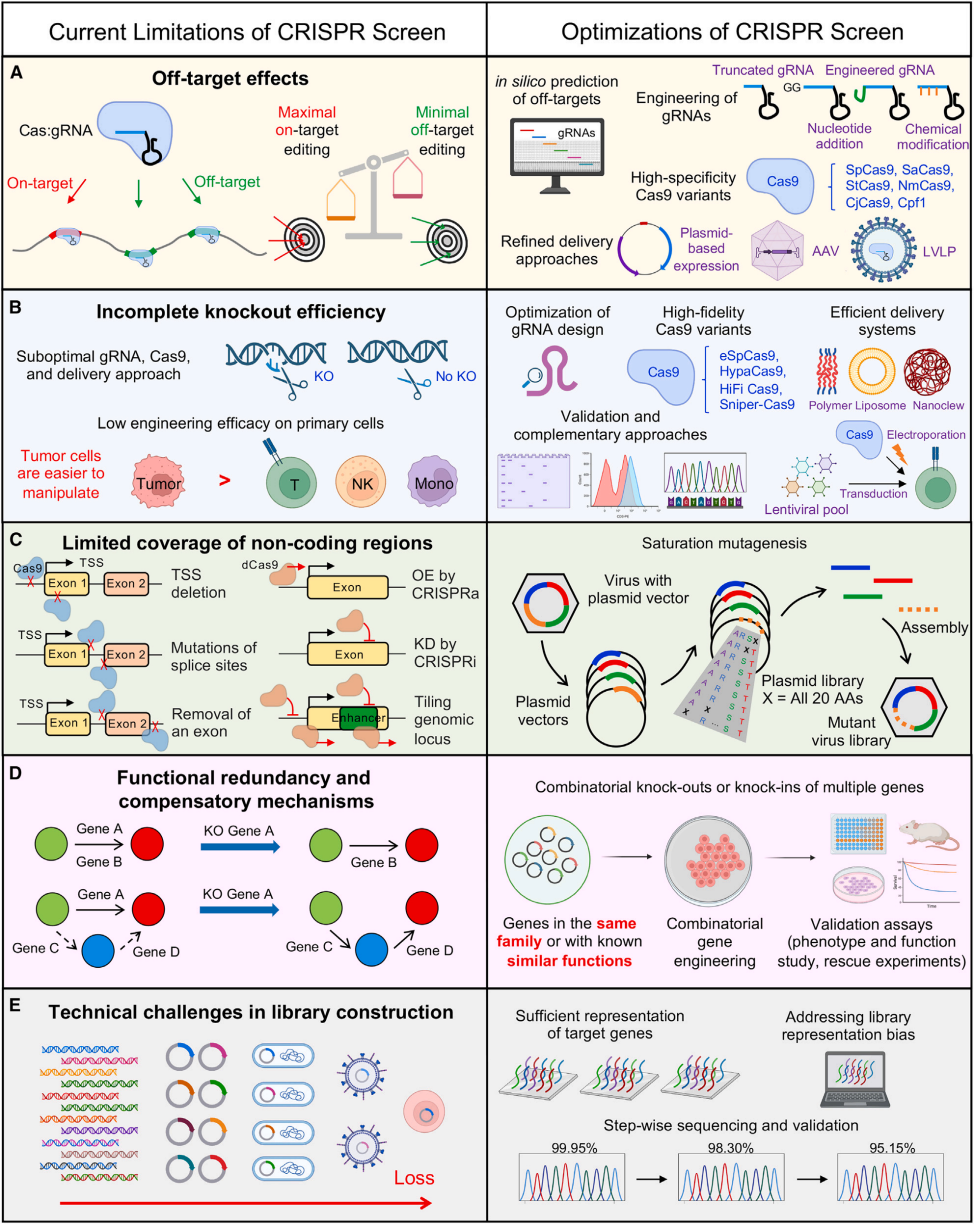
Current limitations and optimizations of CRISPR screen This schematic provides a concise overview of the primary constraints encountered in CRISPR screen technology. These include the propensity for off-target effects resulting from the CRISPR-Cas system (A), incomplete gene knockout efficiency due to the use of suboptimal gRNAs (B), limited coverage of non-coding regions when designing gRNAs (C), the presence of functional redundancy and compensatory mechanisms within certain genes (D), and the technical hurdles associated with library construction (E). Furthermore, the schematic delineates the corresponding remedies proposed for each of these limitations. spCas9, *Streptococcus pyogenes* Cas9; SaCas9, *Staphylococcus aureus* Cas9; StCas9, *Streptococcus thermophilus* Cas9; NmCas9, *Neisseria meningitidis* Cas9; CjCas9, *Campylobacter jejuni* Cas9; Cpf1, class 2 type V CRISPR-associated endonuclease; LVLP, large viral-like particles; KO, knockout; eSpCas9, enhanced *Streptococcus pyogenes* Cas9; HypaCas9, hyper-accurate *Streptococcus pyogenes* Cas9; HiFi Cas9, high-fidelity *Streptococcus pyogenes* Cas9; TSS, transcription start site; OE, overexpression; KD, knockdown; AA, amino acid.

**Figure 4:**
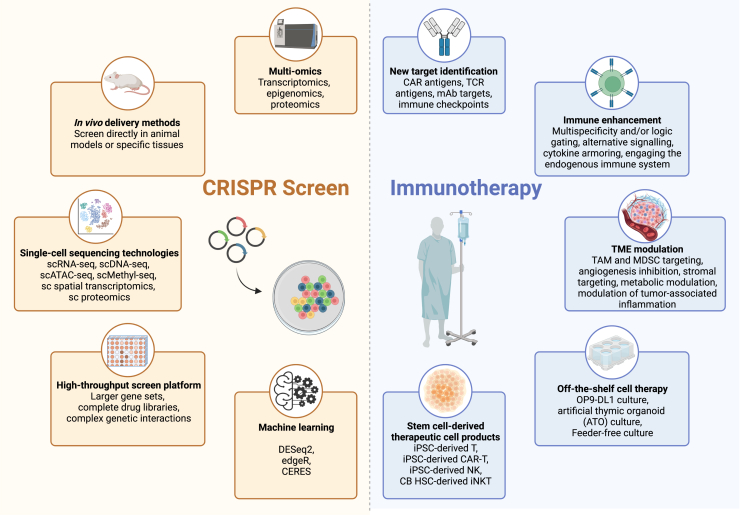
Synergistic integration of other technologies with CRISPR screen for the development of next-generation cancer immunotherapy

In summary, the use of CRISPR screen technology has made substantial strides in enhancing our comprehension of the genetic foundations of cancer immunotherapy. Through systematic manipulation of genes and large-scale screening, this technology has facilitated the identification of crucial genes and pathways involved in anti-tumor immune responses and the emergence of treatment resistance. This review provides a comprehensive overview of the current applications of CRISPR screens in cancer immunotherapy (Figure 1). It emphasizes the use of CRISPR screens in tumor cells for the identification of novel therapeutic targets and the use of CRISPR screens in therapeutic cells to advance cell-based therapies (Figure 2). Furthermore, the review addresses the existing limitations of CRISPR screen technology and explores future research directions in this field (Figures 3 and 4).

## CRISPR screens in tumor cells to identify new therapeutic targets

The use of CRISPR screens in tumor cell lines has provided a rapid and comprehensive approach to investigate numerous genes, enabling the identification of key regulators within extensively studied signaling networks. These networks encompass crucial biological processes such as antigen presentation, IFN-γ signaling, TNF-α signaling, natural killer (NK) cytotoxicity sensitivity, and macrophage recruitment (Table 1). Furthermore, these screens have facilitated the discovery of previously unknown pathways associated with antigen presentation. Here we summarize the novel biological insights garnered from these CRISPR screens and discuss the prospective avenues they have illuminated for future research directions in this field.

**Table 1:** CRISPR screens in tumor cells to identify new targets for cancer immunotherapy

Species	Target cells	Loss or gain of function	CRISPR library	Transduction methods	Selection methods	Genes identified	Corresponding proteins	Gene/protein functions	Years and references
Mouse	melanoma cell line B16	loss of function	self-designed a library of 9,992 optimized sgRNAs targeting 2,398 genes	lentivirus transduction	*in vivo* tumor growth	*Ptpn2*	PTPN2	Deletion of the protein tyrosine phosphatase PTPN2 in tumor cells increased the efficacy of immunotherapy by enhancing interferon-γ-mediated effects on antigen presentation and growth suppression.	Manguso et al. (2017)^43^
	TNBC cell line 4T1	loss of function	MusCK	lentivirus transduction	*in vivo* tumor growth	*Cop1*	COP1	Deletion of Cop1 inhibits macrophage infiltration, macrophage-associated chemokine secretion, and macrophage chemoattractant gene expression, therefore strengthening TNBC sensitivity to ICB.	Wang et al. (2021)^44^
	TNBC cell line 4T1	loss of function	self-designed DrIM and mini-DrIM library (two step)	lentivirus transduction	*in vivo* immune selection between immunocompetent and immunodeficient mice	*Lgals2*	LGALS2	Lgals2 induces proliferation of TAMs, and polarization of macrophages toward M2 through the CSF1/CSF1R pathway, thereby strengthening the immunosuppressive nature of the TNBC TME. Inhibition of LGALS2 suppresses tumor growth.	Ji et al. (2022)^45^
	macrophage-like RAW264.7	loss of function	self-designed a 10 sgRNA-per-gene CRISPR deletion library targeting 1647 RBP genes using CRISPR-FOCUS	lentivirus transduction	*in vitro* PD-L1 expression under IFN-γ stimulation	*Denr*	DENR	Knockout of DENR weakens JAK2 translation and the IFN-γ-JAK-STAT pathway, thereby reducing PD-L1 expression, inhibiting tumor growth, and increasing sensitivity to CD8^+^ T cell cytotoxicity.	Chen et al. (2022)^46^
	renal carcinoma cell line Renca, melanoma cell line B16, breast carcinoma cell lines 4T1 and EMT6, colorectal carcinoma cell lines CT26 and MC38	loss of function	self-designed mTKO library, analogous to human TKOv3 library	lentivirus transduction	*in vitro* sensitivity or resistance to CTL-mediated cytotoxicity	*Fitm2* and a set of autophagy-related genes.	FITM2	Knockout of Fitm2 increases tumor cell sensitivity to CTL-produced IFN-γ through increasing their susceptibility to oxidative proteotoxic and lipotoxic stress. Knockout of autophagy-related genes enhances tumor sensitivity to TNF-mediated cytotoxicity.	Lawson et al. (2020)^47^
	colon adenocarcinoma cell line MC38	loss of function	mouse v2 CRISPR library	lentivirus transduction	*in vitro* sensitivity or resistance to T-cell-mediated cytotoxicity	*PRMT1* and *RIPK1*	PRMT1, RIPK1 (receptor-interacting serine/threonine-protein kinase 1)	PRMT1 and RIPK1 are identified as a dual immune resistance regulator and a cytotoxicity resistance regulator.	Hou et al. (2020)^31^
	B16F10 melanoma cell line	loss of function	mouse CRISPR Brie lentiviral pooled libraries	lentivirus transduction	*in vitro* sensitivity or resistance to T-cell-mediated cytotoxicity	*Pbrm1*, *Arid2*, and *Brd7*	Proteins of the PBAF form of the SWI/SNF chromatin remodeling complex	Loss of PBAF function increased tumor cell sensitivity to IFN-γ, resulting in enhanced secretion of chemokines that recruit effector T cells.	Pan et al. (2018)^48^
Human	Melanoma cell line Mel624	Loss of function	GeCKOv.2	Lentivirus transduction	*In vitro* sensitivity to NY-ESO-1^+^ CD8^+^ T cells	*APLNR*	APLNR (apelin receptor)	Deletion of APLNR inhibits the JAK-STAT pathway, thereby weakening IFN-γ response and sensitivity to T-cell-based immunotherapies	Patel et al..,^49^
	melanoma cell line A375	gain of function	SAM v1	lentivirus transduction	*in vitro* sensitivity to NY-ESO-1^+^ CD4^+^ and CD8^+^ T cells	*CD274*, *MCL1*, *JUNB*, *B3GNT2*	CD274, MCL1, JUNB, B3GNT2	Overexpression of MCL1 and JUNB downregulates the mitochondrial apoptosis pathway for cytotoxicity by inhibiting mitochondrial outer membrane permeabilization and upregulating the NF-κB pathway respectively. Overexpression of B3GNT2 interferes the interactions between T cells and tumor cells, thereby inhibiting T cell activation.	Joung et al. (2022)^38^
	melanoma cell line D10	loss of function	GeCKOv.2	lentivirus transduction	*in vitro* sensitivity to NK and CD8^+^ T cells	*RNF31*	RNF31	Inhibition of RNF31, an E3 ubiquitin ligase, disrupts the cell-ligand-bound TNF receptor complex 1, causing the loss of A20 and non-canonical IKK complexes. Therefore, tumor apoptosis is promoted via the downregulated NF-κB pathway. Pharmacologic inhibition of RNF31 also promotes the bystander killing of tumor cells lacking MHC.	Zhang et al. (2022)^50^
	GSC cell lines: 387, CW468, D456, 1517	loss of function	Brunello	lentivirus transduction	*in vitro* sensitivity to NK cells	*CHMP2A*	CHMP2A	Knockout of CHMP2A upregulates the NF-κB pathway in tumor cells, increasing chemokine secretions to promote NK cell migration and reducing NK cell apoptosis, thereby increasing NK-cell-mediated cytotoxicity.	Bernareggi et al. (2022)^51^
	pancreatic cell lines HupT3 and KP4_MSLN	loss of function	Brunello	lentivirus transduction	*in vitro* sensitivity to MSLN-CAR-T cells	*GPAA1*, *RELA*, *CHUK*, *FADD*, *TFAP4*	GPAA1,RELA, CHUK, FADD, TFAP4	Inhibition of genes involved in GPI anchor biosynthesis pathway increases pancreatic tumor cells resistance to MSLN CAR-T cell therapy. Genes involved in the death receptor pathway sensitizes pancreatic ductal adenocarcinoma to CAR-T-cell-mediated cytotoxicity. TFAP4 loss promotes p65 (NF-κB transcription factor) activity.	Hagel et al. (2023)^52^
	SCLC cell lines: H69, SCLC-A, H82, SCLC-N, and three PDX-derived cell lines	loss of function	Saturn V	lentivirus transduction	*in vitro* sensitivity to Cisplatin (GI 20)	*XPO1*	XPO1	Inhibition of XPO1 increases SCLC sensitivity to chemotherapy. Possible mechanisms include the repression of AKT/mTOR activation.	Quintanal-Villalonga et al. (2022)^53^
	CD19^+^ B-ALL cell lines Reh, NALM6, and 697, and mature B cell neoplasm HG3 and TMD8	loss of function	genome-wide: Brunello; self-designed two pooled libraries targeting CD19 activators and the repressor using https://www.benchling.com/	lentivirus transduction	expression level of CD19	*ZNF143*, *NUDT21*	ZNF143, NUDT21	ZNF, a transcriptional activator, activates CD19 promoter. NUDT21, an RNA-binding protein, suppresses CD19 expression by regulating mRNA stability polyadenylation.	Witkowski et al. (2022)^54^
	human CML cell line K562	loss of function	GeCKO V2	lentivirus transduction	*in vitro* sensitivity to NK cells	*NCR3LG1*, *BCL-ABL*	NCR3LG1, BCL-ABL	Loss of NCR3LG1, which encodes the ligand of the natural cytotoxicity receptor NKp30, protected K562 cells from killing; knocking out BCL-ABL in antigen-presentation pathway and the IFNGR-JAK-STAT pathway increased the vulnerability of K562 cells to NK-cell-mediated lysis.	Zhuang et al. (2019)^33^
	HCT15, SW620, HT29	loss of function	GeCKO V2; Brunello	lentivirus transduction	*in vitro* sensitivity to NK cells	*NCR3LG1*; *HLA-E*	NCR3LG, HLA-E	NCR3LG promotes NK sensitivity; HLA-E suppresses NK sensitivity of tumor cells.	Sheffer et al. (2021)^55^
	AML cell lines MOLM13 and MOLM14	loss of function	GeCKO V2	lentivirus transduction	*in vitro* sensitivity to NK cells	*TNFRSF1B*	TNFRSF1B	A less differentiated phenotype of AML cells confers resistance to NK cell cytotoxicity through lack of TNFRSF1B expression.	Dufva et al. (2019)^56^
	glioblastoma cell line U87	loss of function	a library of more than 76,000 guides targeting around 19,000 genes to induce insertion–deletion mutations (indels)	lentivirus transduction	*in vitro* sensitivity to EGFR-CAR-T cells	*JAK2*, *IFNGR1*, *IFNGR2*	JAK2, IFNGR1, IFNGR2	The loss of genes in the IFN-γR signaling pathway rendered solid tumors more resistant to killing by CAR-T cells.	Larson et al. (2022)^32^
	leukemia monocytic cell line THP-1	loss of function	hCRISPRi v2	lentivirus transduction	STING expression level under CDN stimulation	*SLC19A1*	SLC19A1	Depletion of SLC19A1 inhibits CDN uptake and functional responses, thereby inhibiting cGAS-STING pathway and downstream transcription factors IRF3 and NF-κB.	Luteijn et al. (2019)^57^
	melanoma cell line D10	loss of function	GeCKO	lentivirus transduction	*in vitro* sensitivity to MART-1 T cells	*TRAF2*	TRAF2	Inhibition of TRAF3 redirects TNF signaling pathway to promote RIPK1-dependent apoptosis, thereby decreasing TNF cytotoxicity threshold in tumors.	Vredevoogd et al. (2019)^58^
	AML cell lines MOLM-13, MV4-11, HL-60, OCI-AML2, OCI-AML3	loss of function	a human genome-wide CRISPR library (v1) consisting of 90,709 gRNAs targeting a total of 18,010 genes	lentivirus transduction	AML-specific vulnerability	*KAT2A*	KAT2A	KAT2A inhibition induces myeloid differentiation and apoptosis, and KAT2A inhibition arrests the growth of primary AML cells.	Tzelepis et al. (2016)^59^

CDN, cyclic dinucleotide; CHMP2A, charged multivesicular body protein 2A; CML, chronic myelogenous leukemia; DENR, density-regulated protein; GSC, glioblastoma stem cell; FITM2, fat storage-inducing transmembrane protein 2; IFNGR1, interferon-gamma receptor 1; JAK2, Janus kinase 2; KAT2A, lysine acetyltransferase 2A; LGALS2, galectin-2; NCR3LG1, natural killer cell cytotoxicity receptor 3 ligand 1; NUDT21, nudix hydrolase 21; PRMT1, protein arginine methyltransferase 1; PTPN2, protein tyrosine phosphatase, non-receptor type 2; RNF31, ring finger protein 31; SLC19A1, folate-organic phosphate antiporter; TNFRSF1B, TNF receptor superfamily member 1B; TRAF2, TNF receptor-associated factor 2; TNBC, triple-negative breast cancer; XPO1, exportin 1; ZNF143, zinc finger protein 143.

### Regulators of antigen presentation

Major histocompatibility class I (MHC I) molecules play a crucial role in presenting tumor self-protein peptides on the cell surface, which allows antigen-presenting cells (APCs) to internalize them and activate cytotoxic T cells (CTLs) that specifically target tumor-specific or tumor-associated proteins.^60^ Subsequently, primed CTLs can recognize tumor cells expressing the abnormal protein via MHC I and mount cytotoxic responses against them.^61^ However, tumor cells have developed various mechanisms to circumvent antigen presentation pathways or downregulate the expression of MHC I molecule.^60^ Therefore, it is essential to identify regulator proteins that could potentially disrupt or enhance the antigen presentation in tumor cells, accordingly inhibiting or overexpressing genes encoding those proteins to achieve a more robust CTL-mediated anti-tumor response. CRISPR screen, as a functional and efficient gene editing and assessment tool, has been widely used to explore new antigen-presentation regulators in tumor cells.

Several studies have focused on genes directly encoding the MHC I molecule and controlling its localization on the cell surface. Patel et al.^49^ illustrated that KO of essential MHC I genes, TAP2 and B2M, stimulated tumor cell evasion from T-cell-mediated killing. Similarly, in an *in vivo* screening of genes involved in multiple classes including antigen processing and presentation, cell surface localization, and chromatin remodeling, Manguso et al.^43^ demonstrated that in addition to Tap1 and Tap2, KO of H2-T23, a non-classical MHC I gene contributing to the inhibitory regulation of T cells, also enhanced the effect of immune cytotoxicity. Moreover, other studies have investigated the function of protein-coding genes associated with antigen-processing and presentation processes, despite not being part of the MHC I molecule itself. For instance, an *in vitro* loss-of-function screening determined that inhibition of BCL-ABL in the antigen presentation pathway increased the vulnerability of human chronic myelogenous leukemia (CML) cells to NK-cell-mediated lysis.^33^ Manguso et al.^43^ also illustrated that *Ptpn2* KO increases the level of MHC I on tumor cell surfaces.

In conclusion, most studies examining the effects of antigen presentation mechanisms on immune cell-mediated killing have primarily focused on genes directly encoding MHC I subunits or those involved in regulatory pathways. Although the functions of several MHC I subunits, both canonical and non-canonical, have been extensively studied (TAP1/2, B2M, and H2-T23),^43^^,^^49^ there is still a lack of studies investigating genes indirectly related to MHC I expression and localization (BCL-ABL and Ptpn2),^33^^,^^43^ which presents promising avenues for future screens.

### Regulators of IFN-γ signaling

IFN-γ signaling pathway orchestrates a series of anti-tumor responses, including anti-proliferative and pro-apoptotic effects, regulation of antigen presenting, elevation of inflammatory signals in tumor cells.^62^ Although given the versatile anti-tumor effects of IFN-γ, immunotherapies targeting on IFN-γ have been extensively investigated, they have confronted limitations in achieving widespread success because of the complex interactions of IFN-γ with other proteins in the tumor microenvironment (TME).^63^ Ongoing studies have continually used CRISPR screen to explore the specific function of individual gene that cross-talks with the IFN-γ signaling pathway.

To explicate the role of IFN-γ in PD-L1 expression regulation, Chen et al.^46^ performed an *in vitro* CRISPR screen with a lentivirus single-guide RNA (sgRNA) library targeting RNA-binding proteins (RBPs), indicating that inhibition of *DENR* undermined JAK2 translation and the IFN-γ-JAK-STAT pathway, which consequently reduced PD-L1 expression and promoted sensitivity to CD8^+^ T cell cytotoxicity. Also studying the IFN-γ-JAK-STAT pathway, Patel et al.^49^ exhibited that KO of *APLNR* inhibited the signaling pathway and thereby weakened IFN-γ response and sensitivity to T-cell-based immunotherapies. Performing an *in vitro* screening with multiple categories of tumor cell lines, Lawson et al.^47^ demonstrated that Fitm2, as a universal regulator in multiple types of cancer, increased tumor cell sensitivity to CTL-produced IFN-γ through increasing their susceptibility to oxidative proteotoxic and lipotoxic stress. Manguso et al.^43^ also supplemented that the promotion of antigen presentation by Ptpn2 deletion indeed relied on increased IFN-γ sensing by tumor cells. However, when focusing on the downstream molecule of IFN-γ, JAK, Park et al.^64^ observed a distinct phenomenon regarding the resistance of tumor cells to T cell cytotoxicity under *Jak1*/*Jak2* KO. In their study, by devising a dual perturbation library designed to target both mutated tumor suppressor genes and immune resistance genes, they revealed that the simultaneous KO of *Jak1*/*Jak2* and tumor suppressor genes such as *Trp53* resulted in an increased resistance to OT-I CD8^+^ T cells during combinatorial antineoplastic drug resistance experiment (CADRE) screening, which suggests the existence of intricate gene interaction.^64^

Together, although IFN-γ has complicated interaction with other components of tumor regulation pathways, CRISPR screening has revealed potent modulators that influence PD-L1 expression, antigen presentation, responses to oxidative stress, and downstream IFN-γ-JAK-STAT pathway.^43^^,^^46^^,^^47^,^49^ Future studies could investigate the cross-talk between IFN-γ and other pro-tumor or anti-tumor regulatory molecules and signaling pathways.

### Regulators of TNF signaling

TNF exhibited dual functions in the TME, either as anti-tumor regulator or as an immunosuppressive cytokine, which stimulates examination on its complex role with CRISPR screen.^65^ In an *in vitro* genome-wide screening, Zhang et al.^50^ demonstrated that inhibition of RNF31, an E3 ubiquitin ligase, disrupted the cell-ligand-bound TNF receptor complex 1, leading to the loss of A20 and non-canonical IKK complexes and ultimately promoted tumor apoptosis. Besides, to elicit the impact of autophagy on anti-tumor responses, Lawson et al.^47^ demonstrated that KO of autophagy-related genes, including Atg12 and Tbk1, enhanced tumor sensitivity to TNF-mediated cytotoxicity. To enhance sensitivity of melanoma cancer to MART-1 T cells, Vredevoogd et al.^58^ illustrated that deletion of TNF receptor-associated factor 2 (TRAF2) redirected TNF signaling pathway to promote RIPK1-dependent apoptosis, thereby lowering TNF cytotoxicity threshold in tumor cells. Moreover, Dufva et al.^56^ investigated the functions of TNF-related apoptosis-inducing ligand (TRAIL) receptors and found that when TNFRSF1B expression was inhibited, a less differentiated phenotype of acute myeloid leukemia (AML) cells conferred resistance to NK cell cytotoxicity.

Although current studies have primarily focused on improving anti-tumor responses through perturbations of TNF receptors (i.e., TNF receptor complex 1, TRAF2, and TRAIL receptors), future investigations should also explore the intracellular downstream signaling cascades.^50^^,^^56^^,^^58^

### Regulators of NK cytotoxicity sensitivity

NK cells, belonging to the innate lymphoid cell family, play a crucial role in cancer therapy, including autologous and allogeneic NK-cell-based immunotherapy. These cells demonstrate a broad tumor reactivity and are particularly involved in eradicating early tumors and controlling metastasis.^66^ Tumor cells commonly express stress-inducing molecules, such as NK ligands, including MHC class I polypeptide-related sequence A (MICA), MICB, and UL16-binding proteins (ULBPs), which selectively activate NK cells through the NKG2D receptor.^66^ However, the presence of NK cells is typically reduced in established tumors because of the elevated expression of inhibitory receptors, such as TIGIT, CD96, and TIM3, on tumor-infiltrating NK cells, leading to a substantial impairment of their anti-tumor functionality.^66^ Moreover, the heterogeneous TME modulates NK cell metabolism within the context of solid tumors, posing a significant constraint on their functionality.^67^ Prior research has demonstrated that inhibiting TIM3 expression in NK cells enhances their cytotoxicity and IFN-γ production, providing a potential strategy to overcome the efficacy limitation of NK-cell-based therapy.^68^ Consequently, it is crucial to further investigate the molecular mechanisms underlying NK-cell-mediated tumor eradication in order to enhance their killing capacity and clinical application.

Currently, the integration of CRISPR screens in tumor cells has facilitated the examination of modified NK sensitivity, the discovery of novel immune checkpoint targets, and the improvement of cell-based therapies. Zhang et al.^50^ conducted parallel genome-wide CRISPR-Cas9 KO screens under NK and CD8^+^ T cell pressure, revealing the involvement of various components, including RNF31, RBCK1, and SHARPIN, in the linear ubiquitination chain assembly complex (LUBAC). This complex holds a pivotal role in TNF signaling, governing cellular survival and death. Inhibition or depletion of RNF31 rendered tumor cells more susceptible to both adaptive and innate immune cells, resulting in heightened apoptosis through reduced NF-κB signaling.^50^ These findings propose that targeting RNF31 could enhance TNF-mediated killing and empower NK cell anti-tumor activity.^50^ In a separate study focusing on human glioblastoma stem cells (GSCs), CHMP2A, a chromatin-modifying protein and constituent of the ESCRT-III complex, emerged as a regulator of NK-cell-mediated cytotoxicity. Using a comprehensive “two cell type” whole-genome CRISPR-Cas9 screening system, the KO of CHMP2A triggered augmented migration of NK cells toward tumor cells and increased secretion of chemokines involved in NK cell migration.^51^ These findings underscore the critical involvement of CHMP2A in mechanisms of immune evasion.^51^ Previous research also investigated genes that influence the susceptibility of leukemia cells to primary NK cell killing using a CRISPR screen with the CML cell line K562.^33^ Loss of NCR3LG1, the ligand for natural cytotoxicity receptor NCR3 (NKp30), protected K562 cells from NK-cell-mediated killing.^33^ Additionally, IFNGR2 was found to be responsible for the upregulation of MHC class I molecule on K562 cells following co-incubation with NK cells.^33^ The results were further corroborated by the observation that decreased expression of IFNGR2 was associated with enhanced overall survival in patients diagnosed with AML and kidney renal clear cell carcinoma (KIRC).^33^ Moreover, NK-cell-sensitive tumor cells were found to exhibit elevated expression of chromatin remodeling complexes, heightened levels of B7-H6 (NCR3LG1), and reduced levels of HLA-E/antigen presentation genes using profiling relative inhibition simultaneously in mixtures (PRISM) phenotypic screens and CRISPR gene-editing studies. The combined use of PRISM and CRISPR provided a “multi-omic” and functional genomic profile of a representative NK-cell-sensitive tumor cell, with implications for NK cell immunotherapies and their correlation with immune checkpoint inhibitor (ICI) resistance.^55^ Furthermore, Dufva et al.^56^ conducted genome-wide CRISPR screens to identify genes associated with antigen presentation, interferon signaling, and various factors such as NCR3LG1, apoptotic mediators, TRAIL receptors, CD48, and TNFRSF1B, which influence the resistance of hematologic cancer cells to NK cell cytotoxicity. The results showed that different lineages of hematological malignancies exhibited distinct susceptibility mechanisms to NK cells, and this variability was determined by the lineage-specific expression of susceptibility genes.^56^ Another study further confirmed the role of CD48 expression in evading NK-cell-mediated immunity in adult T cell leukemia/lymphoma (ATLL) cells. Genome-wide CRISPR screening identified CD48 as a gene whose KO conferred resistance to NK cell cytotoxicity. Reduced CD48 expression was observed in primary ATLL cells and other aggressive peripheral T cell lymphomas, indicating its significance as a biomarker for NK-cell-associated immunotherapies.^69^ In summary, the successful use of CRISPR screens has greatly contributed to the identification of numerous targets for the improvement of NK cell therapy. Future applications of genome-wide CRISPR screens on effector NK cells hold promise in directly elucidating the molecular mechanisms underlying their anti-tumor efficacy.

### Regulators of macrophage recruitment

Macrophages, a type of phagocytic cells, can be classified into M1 and M2 macrophages, each characterized by unique functions. Although classically activated proinflammatory M1 macrophages mainly contribute to the elimination of pathogens and tumor cells through several ways, such as producing nitric oxide, alternatively activated anti-inflammatory M2 macrophages promote the removal of parasites and homeostasis through the high secretion of polyamines and ornithine.^70^ However, these versatile cells play an important role in tumor progression and immunosuppression,^70^^,^^71^^,^^72^ especially M2 tumor-associated macrophages (TAMs) which are the major part of tumor myeloid cells.^70^ By generating various cytokines, such as platelet-derived growth factor (PDGF) and ligands of the epithelial growth factor receptor (EGFR) family, TAM infiltration aggravates tumor development.^73^^,^^74^ Moreover, M2 macrophages destroy the matrix membrane of endothelial cells through soluble factors, including matrix metalloproteinases (MMPs), serine proteases, etc., hence promoting the epithelial-mesenchymal transition (EMT) of tumor cells, which is the foundation of tumor metastasis.^75^^,^^76^^,^^77^ Conversely, tumor cells generate cytokines that enhance the differentiation of TAMs, leading to the ultimate reciprocity between TAMs and tumor cells.^78^ Therefore, it is crucial to figure out the mechanisms behind this positive loop and solve the problems accordingly for better tumor treatment outcomes.

On the basis of the nature of macrophages, macrophage-related genes could be potential targets for cancer immunotherapy. Although blocking the recognized immune checkpoints has proved clinically effective, the limited response to treatment highlights the existence of extra immune escape mechanisms. To further deal with this problem, CRISPR screens have been successfully used to identify additional immune checkpoints. For example, for triple-negative breast cancer (TNBC), results of pooled *in vivo* CRISPR KO screens in syngeneic mouse models have demonstrated that the elimination of the E3 ubiquitin ligase Cop1 in cancer cells could lead to a reduction in the secretion of chemokines associated with macrophages, resulting in decreased infiltration of tumor macrophages.^44^ As a result, targeting Cop1 improves the anti-tumor immune response and strengthens the response to immune checkpoint blockade.^44^ Furthermore, in another study of TNBC treatment, two-step customized *in vivo* CRISPR screens in mouse models with a designed mouse sgRNA library that corresponds to all human disease-related immune (DrIM) genes recognize Lgas2, which promotes the M2-like polarization and macrophage proliferation by activating the colony-stimulating factor 1 (CSF1)/CSF1 receptor (CSF1R) axis, as an important regulation for the progression of TNBC.^45^ These significant findings suggest the potential of CRISPR screens in the improvement of cancer immunotherapy.

## CRISPR screens in immune cells to develop advanced cell therapies

### CRISPR screens in T and CAR-T cells

T-cell-based therapies, particularly chimeric antigen receptor (CAR)-engineered T cell (CAR-T) therapy, have revolutionized the treatment of hematological malignancies and demonstrated remarkable clinical outcomes to date.^79^^,^^80^^,^^81^^,^^82^^,^^83^ CAR antigens, such as CD19, BCMA, CD70, mesothelin, EGFR, and others, have been extensively investigated and applied in various cancer types.^84^^,^^85^^,^^86^^,^^87^^,^^88^ Despite these advancements, CAR-T-cell-based therapy still faces certain limitations, including its limited efficacy in solid tumors, as well as the occurrence of adverse effects such as cytokine release syndrome (CRS) and neurotoxicity.^89^^,^^90^^,^^91^^,^^92^ To overcome these limitations and develop more robust CAR-T cell products, it is imperative to elucidate the underlying molecular mechanisms that enhance anti-tumor efficacy while mitigating safety concerns. In this context, the application of CRISPR screens has emerged as a valuable tool, providing comprehensive and detailed insights into the selection of optimal CAR-T cells. CRISPR screens enable the identification of CAR-T cells with enhanced characteristics, such as accelerated proliferation and division rates, potent anti-tumor activity, reduced exhaustion, and improved tumor infiltration capabilities (Table 2). Leveraging the power of CRISPR technology, we can guide the generation of optimized CAR-T cell products tailored for cancer therapy.

**Table 2:** CRISPR screens in T cells to develop advanced T-cell-based products for cancer immunotherapy

Species	Target cells	Loss or gain of function	CRISPR library	Transduction methods	Selection methods	Genes identified	Corresponding proteins	Gene/protein functions	Years and references
Mouse	CD4^+^ T cells	loss of function	library pMSCV-U6gRNA(lib)-PGKpuroT2ABFP (Addgene: #104861)	retrovirus transduction	expression of IRF4, XBP1, or GATA3	*Pparg* and *Bhlhe40*	PPARG (peroxisome proliferator activated receptor gamma) and BHLHE40 (basic-helix-loop-helix protein 40)	PPARG and BHLHE40 are crucial to TH2 gene regulation and differentiation. Genes regulating TH2 activation and genes regulating TH2 differentiation are highly overlapped.	Henriksson et al. (2019)^93^
	CD8^+^ T cells	loss of function	retroviral mouse genome-wide CRISPR knockout library (Addgene #104861), containing 90,230 sgRNAs with 4 guides per gene	retrovirus transduction	*in vitro* T cell exhaustion assay	*Arid1a*	ARID1A	ARID1A depletion limits the acquisition of exhaustion-associated chromatin accessibility and leads to improved anti-tumor immunity.	Belk et al. (2022)^94^
	CD8^+^ T cells	loss of function	a self-designed sgRNA library targeting exonic regions of 25 kinases showing kinase activity in T cells after TCR stimulation, with three sgRNAs per gene	electroporation	cell expansion, differentiation, oxidative stress, and genomic stress	*Mapk14*	MAPK14 (p38-α)	Low level of MAPK14 improves the efficacy of mouse anti-tumor T cells.	Gurusamy et al. (2020)^95^
	CD8^+^ T cell	loss of function	a self-designed domain-focused sgRNA library against 120 TFs, including 675 sgRNAs in total, with 4–5 sgRNAs per DNA-binding domain, positive selection controls (sgPdcd1), and non-selection controls	retrovirus transduction	cell proliferation	*Fli1*	FLI1	FLI1 depletion enhances effector T cells’ responses without compromising memory or exhaustion precursors whereas high level of FLI1 restrains differentiation. CD8^+^ T cells lacking FLI1 provides substantially better protection against multiple infections and tumors.	Chen et al. (2021)^96^
	CD8^+^ T cells	loss of function	sgRNA Brie library; two lentiviral sub-libraries of sgRNAs (six sgRNAs per gene) targeting 3,017 metabolic enzymes, small molecule transporters and metabolism-related transcriptional regulators	lentivirus transduction	cell proliferation in tumor-infiltrating lymphocytes	*Zc3h12a (Regnase-1)*	REGNASE-1	REGNASE-1-deficient CD8^+^ T cells are reprogrammed in the TME to long-lived effector cells by enhancing BATF function and mitochondrial metabolism, thereby improving adoptive cell therapy for cancer.	Wei et al. (2019)^97^
	CD8^+^ T cells	loss of function	a focused sgRNA library (mouse surface and membrane protein-encoding gene library, Surf) targeting 1,658 membrane-bound protein-coding genes (four sgRNAs were chosen per gene similar to the mBrie library design49), with 6,628 sgRNAs and 1,000 NTCs	AAV transduction	cell proliferation in brain	*Mgat5 and Pdia3*	MGAT5 and PDIA3	Adoptive transfer of CD8^+^ T cells deficient in PDIA3, MGAT5, EMP1, or LAG3 enhances the survival of glioblastoma-bearing mice in both syngeneic and T cell receptor transgenic models.	Ye et al. (2019)^98^
	CD8^+^ T cells	loss of function	a mouse genome-scale sgRNA library (MKO) containing 128,209 gene-specific sgRNAs that target every gene in the genome and 1,000 NTCs	lentivirus transduction	cell number in tumor	*Dhx37*	DHX37	DHX37 modulates CD8 T cell activation, cytokine production, and cytotoxicity. Dhx37 knockout in CD8 T cells enhances adoptive transfer efficacy.	Dong et al. (2019)^99^
	CD8^+^ T cell	loss of function	a self-designed sgRNA library of 110 sgRNAs targeting 21 genes relevant to T cell biology and 50 NTC sgRNAs.	lentivirus transduction	cell proliferation	*Ptpn2*	PTPN2	PTPN2 is a negative regulator of CD8^+^ T-cell-mediated responses to LCMV clone 13 viral infection.	Lafleur et al. (2019)^100^
	CD8^+^ hEAR2 targeting CAR-T cells	loss of function	a self-designed sgRNA library with 5 sgRNAs per gene, targeting 1,316 genes that are expressed differentially in *in vivo* activated T cells and naive T cells	retrovirus transduction	cell number in circulation	*St3gal1*	ST3GAL1	ST3GAL1 is a negative regulator of the tumor-specific CAR-T cell migration.	Hong et al. (2023)^101^
	regulatory T cells	loss of function	a self-designed sgRNA library against 489 targets with 4 guides per gene on the basis of the Brie library to identify gene regulatory programs that promote or disrupt Foxp3 expression	retrovirus transduction	Foxp3 expression	*Usp22* and *Rnf20*	USP22 and RNF20	Usp22 is revealed to be a positive regulator that stabilized Foxp3 expression. Rnf20 can serve as a negative regulator of Foxp3.	Cortex et al. (2020)^102^
Human	CD4^+^ and CD8^+^ T cells	gain of function	a lentiviral library of barcoded human ORFs; nearly 12,000 full-length genes with around 6 barcodes per gene	lentivirus transduction	cell proliferation	*LTBR*	LTBR	When overexpressed in T cells, LTBR induces profound transcriptional and epigenomic remodeling, leading to increased T cell effector functions and resistance to exhaustion in chronic stimulation settings through constitutive activation of the canonical NF-κB pathway.	Legut et al. (2022)^103^
	CD8^+^ T cells	loss of function	pMD2.G (Addgene, catalog #12259) and psPAX2 (Addgene, catalog #12260) containing 77,441 sgRNAs (19,114 genes)	SLICE	cell division tested by CFSE	*DGKA*, *DGKZ*, *TCEB2*, *SOCS1*, *UBASH3A*, *CBLB*, *CD5*, *RNF7*, *CUL5*, *TNFAIP3*, *TNIP1*, and *RASA2*	DGKA, DGKZ, TCEB2, SOCS1, UBASH3A, CBLB, CD5, RNF7, CUL5, TNFAIP3, TNIP1, and RASA2	Cells deficient in identified proteins show a marked increase in number of divisions post stimulation compared with controls.	Shifrut et al. (2018)^35^
	CD8^+^ NY-ESO-1 TCR-specific T cells	loss of function	CRISPR-Cas9 pooled library (rank candidate genes → single gene validation experiment for high ranking genes)	lentivirus transduction	immunofluorescence staining (CD107a^+^) after an exhaustion assay	*SNX9*	SNX9	Depletion of SNX9 enhances memory differentiation, prevents T cell exhaustion, and improves anti-tumor efficacy.	Trifny et al. (2023)^104^
	HA-28z targeting CAR-T cells	loss of function	a self-designed sgRNA library containing 19,885 genes targeted with at least four sgRNAs per gene	SLICE	replicate expansion screen; cytokine production screen	*MED12* and *CCNC*	MED12 and CCNC	Deletion of MED12 or CCNV in human CAR-T cells results in increased proliferation, cytokine production, and increases tumor clearance by reducing steric hindrance between core Mediator and RNAPII.	Freitas et al. (2022)^105^
	CD8^+^ T cells for screening; CD19 targeting CAR-T cells (for validation)	loss of function	the genome-wide Brunello sgRNA library	SLICE	cell proliferation tested by CFSE	*RASA2*	RASA2	RASA2-deficient T cells show increased activation, cytokine production, and metabolic activity in repeated tumor antigen stimulations, and demonstrate an advantage in persistent cancer cell killing.	Carnevale et al. (2022)^106^
	CD8^+^ CAR-T cells	gain of function	a self-designed lentiviral mouse genome-scale dead-guide RNA library (mm10dgLib) using the promoter sequences of all annotated protein-coding transcripts from the mm10 genome assembly. The final mm10dgLib consists of 84,601 dead-guide RNAs that target 22,391 coding transcripts and 1,000 NTCs.	lentivirus transduction	intracellular flow cytometry (CD107^+^) after a kill assay	*PRODH2*	PRODH2	High level of PRODH2 enhances CD8^+^ T cell effector function.	Ye et al. (2022)^107^

AAV, adeno-associated virus; ARID1A, AT-rich interactive domain-containing protein 1A; CCNC, cyclin C; DHX37, DEAH-box helicase 37; FLI1, friend leukemia integration 1 transcription factor; LCMV, lymphocytic choriomeningitis virus; LTBR, lymphotoxin beta receptor; MAPK14, mitogen-activated protein kinase 14; MED12, mediator complex subunit 2; MGAT5, alpha-1,6-mannosylglycoprotein 6-beta-N-acetylglucosaminyltransferase A; NTC, nontargeting control; ORF, open reading frame; PDIA3, protein disulfide isomerase associated 3; PRODH2, proline dehydrogenase 2; PTPN2, protein tyrosine phosphatase nonreceptor type 2; RASA2, RAS p21 protein activator 2; REGNASE-1, regulatory RNase 1; RNF20, ring finger protein 20; SLICE, sgRNA lentiviral infection with Cas 9 electroporation; SNX9, sorting nexin 9; ST3GAL1, ST3 β-galactoside α-2,3-sialyltransferase 1; USP22, ubiquitin-specific peptidase 22.

Unlike immortal tumor cells, the efficiency of transduction and genetic engineering is often hindered in primary cells, which are non-immortalized and have limited expansion potential in culture. Consequently, conducting large-scale pooled screens in primary cells becomes challenging.^108^ To address this issue, researchers proposed a hybrid system that combines lentivirus-mediated introduction of traceable sgRNA cassettes with electroporation of Cas9 protein.^35^ This approach, known as sgRNA lentiviral infection with Cas9 protein electroporation (SLICE), offers several advantages for studying gene function and conducting genome-wide screens in primary cells. First, it enables efficient and specific disruption of target genes, allowing researchers to elucidate their roles in various biological processes. Second, the use of traceable sgRNA cassettes facilitates the tracking and identification of cells in which gene disruption has occurred. This feature is particularly valuable when studying complex cellular processes or conducting pooled screens, as it allows the identification of cells that exhibit specific phenotypic changes resulting from gene disruption. Finally, SLICE can be applied to a wide range of primary cell types, allowing investigations into diverse biological systems and diseases.^35^^,^^108^

A variety of selection indicators have been applied to T cells and CAR-T cells. *In vitro* indicators include the proliferation,^35^^,^^95^^,^^96^^,^^100^^,^^103^^,^^105^^,^^106^^,^^108^ protein expression level,^93^^,^^102^^,^^104^^,^^107^ cytokine production level,^105^^,^^94^ tumor killing efficacy,^107^ differentiation,^95^ oxidative stress,^95^ and genomic stress.^95^ Meanwhile, *in vivo* screens have been carried out to provide useful information regarding the migratory tendency of T and CAR-T cells by measuring their cell number in tumor-infiltrating lymphocytes (TILs),^97^ tumors,^98^^,^^99^ and circulation.^101^ Some projects have applied multiple screens and considered several phenotypic qualities together.^95^^,^^105^ The combination of multiple selection indicators may lead to more accurate and powerful screens in the future.

Up to now, numerous studies have identified essential genes and proteins to improve the functions and anti-tumor reactivity of T and CAR-T cells: PPARG and BHLHE40 are related to TH2 gene regulation and differentiation^93^; ARID1A,^94^ SNX,^104^ and LBTR^103^ are related to exhaustion resistance; MAPK14,^95^ FLI1,^96^ MCAT5 and PDIA3,^102^ RASA2,^106^ PRODH2,^107^ and MED12 or CCNV^105^ are related to anti-tumor efficacy; REGNASE-1 is related to BATF function^97^; DHX37 is related to T cell activation and cytokine production^93^; PTPN2 is related to CD8^+^ T-cell-mediated responses to viral infection^100^; ST3GAL1 is related to the tumor-specific CAR-T cell migration^101^; USP22 and RNF20 are related to stabilized expression of Foxp3; and DGKA,^35^ ACTR6, and RCOR1^108^ are related to cell division.

Moreover, it is important to acknowledge the heterogeneity observed among different cancer types. For example, CAR-T cell killing requires the IFN-γR pathway in solid but not liquid tumors.^32^ By using diverse cancer models as selection approaches, we can specifically design CAR-T cells targeting particular cancers, thereby enhancing both the targeting specificity and therapeutic efficacy of CAR-T-cell-based therapies. To date, only a small number of screens have used cancer model during selection process, focusing on melanoma, glioblastoma, and breast cancer.^97^^,^^98^^,^^99^^,^^107^ In the future, models of more cancer types are to be explored to gain a complete landscape of cancer immunotherapy. At the same time, it is worth noting that all the screens including cancer models use cell lines to carry out kill assays and other assays. Considering the differences between primary cell culture and cell lines, incorporation of primary tumor cells in future screenings may achieve a more accurate simulation of the interaction between immune cells and tumor cells, thus advancing our understanding of cancer therapy.

### CRISPR screens in NK cells

NK cells have emerged as important players in cancer immunotherapy, offering unique capabilities to recognize and eliminate tumor cells without prior sensitization.^66^ However, several limitations hinder their therapeutic potential, including difficulties in infiltrating solid tumors,^109^ alterations in NK activating receptors and ligands within the tumor environment,^110^ and their limited lifespan. To overcome these challenges, CRISPR screen techniques have been used to unravel the intricate interactions between NK cells, tumors, and other immune cells, leading to the development of more effective NK cell products. Current CRISPR screens in NK cells primarily use lentivirus transduction and Cas RNP nucleofection methods (Table 3). For instance, using IL-2-dependent NK cell line NK-92 and a self-designed CRISPR library, Huang et al.^111^ identified the activation of *FCGR3A* and *CD226* genes enhanced the cytotoxicity of NK cells. In a recent study, Peng et al.^112^ performed perturbomics mapping of tumor-infiltrating NK cells by *in vivo* adeno-associated virus (AAV)-CRISPR screens in four distinct mouse models of melanoma, breast cancer, pancreatic cancer, and glioblastoma. The CRISPR screens identified *CALHM2*, a regulator of calcium homeostasis, and showcased substantial efficacy enhancements both *in vitro* and *in vivo* upon perturbing *CALHM2* in CAR-engineered NK cells.^112^

**Table 3:** CRISPR screens in other immune cells to enhance their anti-tumor reactivity

Species	Target cells	Loss or gain of function	CRISPR library	Transduction methods	Selection methods	Genes identified	Corresponding proteins	Gene/protein functions	Years and references
Mouse	raw macrophage cell line 264.7	loss of function	self-designed library with 7272 sgRNA targeting classical RBP genes	lentivirus transduction	TNF-α production	*Mettl3*	METTL3	METTL3-mediated m6A modification of Irakm mRNA accelerates its degradation, resulting in reprogramming macrophages for activation.	Tong et al. (2021)^113^
	cDC1s	loss of function	self-designed functional CRISPR library	retrovirus transduction	OVA cross-presentation	*Wdfy4*	WDFY4	Loss of function of BEACH domain–containing protein Wdfy4 substantially impaired cross-presentation of cell-associated antigens by cDC1s in mice.	Theisen et al. (2018)^114^
Human	IL-2 dependent NK cell line NK-92	gain of function	self-designed library with 22 gRNAs using Benchling’s CRISPR Design tool	Cas RNP nucleofection	calcein-AM cytotoxic assay	*FCGR3A*	FCGR3A	Cas9-mediated promoter insertion effectively reactivated the endogenous FCGR3A and CD226 enhanced NK-92 cytotoxicity.	Huang et al. (2020)^111^
						*CD226*	CD226		
	moDC	loss of function	Brunello	electroporation of *in vitro*-assembled Cas9-sgRNA complexes	loss of TNF-α secretion	*TLR2*	TLR2	*TLR2* knockout caused decrease in TNF-α secretion.	Jost et al. (2021)^115^
						*PTPN6*/*SHP-1*	PTPN6	*PTPN6* knockout strongly increased TNF-α secretion and moderately decreased IL-10 secretion.	
	human myeloid cell line U937	loss of function	self-designed 10-sgRNA-per-gene CRISPR-Cas9 deletion library	lentivirus transduction	phagocytosis ability	*NHLRC2*	NHLRC2	NHLRC2 negatively regulates RHOA, enabling RAC1-mediated cytoskeletal changes that are critical for phagocytosis.	Haney et al. (2018)^116^
						*ELOVL1*	ELOVL1	*ELOVL1* gene disruption causes significant decrease in phagocytosis.	
						*TM2D3*	TM2D3	TM2D2- and TM2D3-deficient cells show impaired clearance of amyloid-β aggregates.	

cDC1, classical dendritic cell; ELOVL1, elongation of very long chain fatty acids protein 1; FCGR3A, Fc gamma receptor IIIa; IL-2, interleukin-2; METTL3, methyltransferase 3; moDC, monocyte-derived dendritic cell; NHLRC2, NHL repeat containing protein 2; PTPN6, protein tyrosine phosphatase non-receptor type 6; TLR2, Toll-like receptor 2; TM2D3, TM2 domain containing 3; WDFY4, Wdfy family member 4.

### CRISPR screens in other immune cells

In addition to NK cells, macrophages, dendritic cells (DCs), and monocyte-derived DCs (moDCs) contribute significantly to tumor targeting and immunosurveillance (Table 3). Macrophages phagocytose tumor cells and stimulate immune responses through antigen presentation, while DCs and moDCs capture and present tumor antigens to activate T cells. These immune cells work synergistically to mount effective anti-tumor responses, highlighting the importance of understanding their interactions for comprehensive cancer immunotherapy strategies. CRISPR screen studies using lentivirus transduction have focused primarily on TNF-α secretion pathways in macrophages and moDCs, revealing the roles of METTL3-mediated m6A modification of Irakm mRNA and the KO of TLR2 and PTPN6.^113^^,^^115^ DC studies, using retrovirus transduction, have emphasized antigen cross-presentation, uncovering the impact of Wdfy4 loss on cDC1s in mice.^114^ However, challenges persist, such as the absence of sgRNA barcodes for pooled screens in DCs. This can be addressed by adapting lentiviral sgRNA delivery and Cas9 electroporation for pooled screening. Additionally, targeting highly homologous genes with CRISPR can be improved through enhanced sgRNA design using computational tools and exploring alternative genome editing techniques. Resolving these limitations will contribute to a better understanding of immune cell interactions and facilitate the development of comprehensive cancer immunotherapy strategies.^114^

## Current limitations and optimizations of CRISPR screen

### Off-target effects

CRISPR screens are prone to unintended genetic modifications resulting from off-target activity of the CRISPR-Cas system, leading to false-positive or false-negative outcomes.^117^ In the case of active Cas9, off-target activity at perfectly matched sites or sites with 1 or 2 mismatches has been observed to impair cell fitness and complicate gene-targeting growth screens.^118^^,^^119^^,^^120^^,^^121^ However, the impact of off-target activity on gene-targeting growth screens is believed to be minimal for CRISPR interference (CRISPRi) or CRISPR activation (CRISPRa).^122^ CRISPRi and CRISPRa are transcription regulation model that use nuclease-dead Cas9, a nuclease-deficient variant of the Cas9 protein. dCas9 has the same efficiency as Cas9 in binding to specific genomic regions, but it is incapable of creating a double-stranded break at the binding site. In practice, dCas9, when fused with transcriptional repression or activation domains, is recruited to the transcription start site of the target gene to either repress or activate its transcription. Essentially, CRISPRi and CRISPRa introduce reversible transcriptional control without genetically altering the target sequence by recruiting transcription factors artificially. Furthermore, off-target effects may pose more challenges in non-coding screens compared with gene screens.^123^

Advancements in CRISPR technology aim to enhance specificity and reduce off-target effects through the implementation of improved design strategies and delivery methods. This entails the development of more precise gRNA design algorithms, Cas variants with diminished off-target activity, refined delivery approaches, and exploration of alternative CRISPR enzymes that do not strictly require a protospacer-adjacent motif (PAM).^121^^,^^123^^,^^124^^,^^125^^,^^126^^,^^127^^,^^128^^,^^129^ We anticipate that the combination of these technological enhancements, thoughtful screen design, and meticulous data analysis considering guide specificity will facilitate comprehensive functional characterization of essential regulatory elements in CRISPR screens^123^^,^^130^ (Figure 3A).

### Incomplete KO efficiency

CRISPR screens may exhibit incomplete KO efficiency, which can arise from various factors, including suboptimal gRNA design, limited Cas9 activity, inefficient delivery of CRISPR components, or inherent biological constraints in achieving complete gene disruption.^131^^,^^132^ The consequence of incomplete KO efficiency is the potential masking or underestimation of the functional impact of the targeted gene, leading to possible false-negative outcomes in the screen.

To address the issue of incomplete KO efficiency, researchers use several strategies. Optimizing gRNA design to enhance targeting specificity and efficiency is a common approach.^12^^,^^130^ Using high-fidelity Cas9 variants with improved editing precision and using delivery methods that ensure effective transfer of CRISPR components into the target cells are also essential.^125^^,^^133^ Enhancing the efficiency of CRISPR screening can be achieved by engineering the target cells to express the CRISPR-Cas protein, allowing ectopic delivery of only the gRNAs during the screen.^12^ The inherent low efficiency of large-scale pooled screens in non-immortalized primary cells (e.g., T cells, NK cells, monocytes), which have limited culture expansion potential, posed a significant hurdle.^35^ To overcome this limitation, a hybrid system was developed using lentivirus-mediated delivery of traceable sgRNA cassettes, followed by electroporation with Cas9 protein, enabling efficient genetic perturbation in primary immune cells.^35^^,^^134^ Validation experiments play a crucial role in mitigating incomplete KO efficiency. Lentiviral transduction of gRNAs targeting test loci,^135^^,^^136^ followed by evaluating editing efficiency at the DNA level using targeted DNA sequencing, or at the protein level using western blotting or flow cytometry, can provide valuable insights.^137^^,^^138^ Furthermore, using complementary approaches, such as RNAi or small-molecule inhibitors, serves to validate the functional relevance of candidate genes identified through CRISPR screens.^139^ Addressing incomplete KO efficiency is of utmost importance to ensure the accuracy and reliability of CRISPR screens, as it directly influences the interpretation of gene functions and their impact on cellular processes or disease phenotypes (Figure 3B).

### Limited coverage of non-coding regions

More than 98% of human genes consist of noncoding regions, which have been discovered to play a crucial role in gene expression regulation and are associated with 90% of diseases and trait-associated variants.^140^^,^^141^ Currently, in adoptive immunotherapy, CRISPR screens predominantly focus on protein-encoding genes, while the noncoding regions remain relatively unexplored, which results in an incomplete capture of the genetic factors that influence the proliferation, infiltration, and cytotoxicity of immune cells, including CD8^+^ T cells and NK cells. There are technical challenges involved in conducting effective CRISPR screens on the noncoding genome. The primary issue pertains to the construction of sgRNA libraries. Given the vast size of the noncoding genome, no available method exists for genome-wide, unbiased, and saturated perturbation. Current techniques either enable saturation of a specific locus through CRISPR perturbations or target pre-identified regions on the basis of specific genomic features.^142^ Additionally, some non-coding regions are relatively small, such as 5–10 bp transcription factor binding sites, requiring precise mutagenesis at specific locations.^143^^,^^144^ Without precise mutagenesis sites, CRISPR screens can only attribute regulatory functions to extended non-coding regions, while the detailed regulatory mechanisms within each region remain unknown.

Despite these limitations, CRISPR screening, as a high-throughput method, serves as an exceptional tool for the functional characterization of the noncoding genome. Future efforts could be directed toward developing improved CRISPR libraries that facilitate large deletions and genetic interaction studies by using paired sgRNA.^144^ Furthermore, the integration of powerful technologies such as single-cell sequencing, cytometry by time of flight (CyTOF), and cellular barcoding into the screening process holds great potential^144^ (Figure 3C).

### Functional redundancy and compensatory mechanisms

Genes involved in complex biological processes often exhibit functional redundancy or compensatory mechanisms, in which genes have homologous counterparts that perform identical or analogous functions within the genome, or the expression of alternative proteins in the pathways is stimulated.^145^^,^^146^ Consequently, when conducting CRISPR screens to knock out or activate a single gene, the complete functional impact may not be fully understood, as other genes can compensate for the loss or gain of function.^147^^,^^148^

Several methods have been demonstrated to mitigate the redundancy or compensation effects, thereby improving the reliability and efficiency of CRISPR screening. Validating the screening outcomes after identifying gene candidates is the initial crucial step in preventing potential effects, as it confirms the exclusive influence of perturbing the candidate gene on phenotypes.^149^ This validation process can be achieved through independent CRISPR disruption, confirmation at the protein level, and subsequent rescue experiments.^150^^,^^151^ Furthermore, an additional vital approach to avert compensatory effects is to implement combinatorial KOs or knockins of multiple genes in the same family or those with known similar functions, which can be accomplished by simultaneously delivering multiple sgRNAs targeting different genes.^152^^,^^153^^,^^154^ By using these diverse approaches, researchers can enhance the robustness of CRISPR screening, enabling a comprehensive understanding of gene function and its impact on phenotypes (Figure 3D).

### Technical challenges in library construction and delivery

Generating high-quality CRISPR libraries and effectively delivering them to target cells present technical challenges that can affect the reproducibility and outcomes of screening experiments. The variability in library construction and delivery methods contributes to these challenges. One specific challenge in CRISPR screens is the capture of gRNAs.^12^^,^^155^ Traditional single-cell RNA sequencing (scRNA-seq) methods rely on polyadenylation to capture and amplify mRNA molecules, but gRNAs lack this polyadenylation.^156^^,^^157^^,^^158^ To overcome this, two approaches can be considered. First, modifying gRNAs by adding a polyadenylated tail allows their capture. Techniques such as perturbation sequencing (Perturb-seq) and CRISPR droplet sequencing (CROP-seq) use this strategy to link gRNAs to polyadenylated transcripts expressed by polymerase II.^154^^,^^159^^,^^160^^,^^161^ Alternatively, gRNAs can be directly captured and amplified, enabling the assessment of their expression and its association with the expression of target genes.^161^^,^^162^

Another challenge in CRISPR library construction is ensuring library complexity. Designing a comprehensive and diverse gRNA library requires careful consideration to cover the entire genome or specific gene sets of interest. Precise design and optimization are essential to ensure sufficient representation of target genes. Additionally, addressing library representation bias, where certain gRNAs are overrepresented or underrepresented, is crucial to avoid potential biases in the screening results. Overcoming these challenges is essential to achieve accurate and comprehensive outcomes in CRISPR screens^163^ (Figure 3E).

## Synergistic integration of cutting-edge technologies with CRISPR screen

In the realm of CRISPR screen, the advent of base editors and prime editors has heralded a transformative era. Base editors, distinguished by their surgical precision, facilitate an in-depth exploration of gene function by instigating specific point mutations. This capability permits the dissection of nucleotide-level contributions to phenotypic variations.^164^ Conversely, prime editing empowers researchers to conduct intricate genome-wide screens, offering the precise modification of genes, promoters, and non-coding elements with unmatched efficiency and minimal off-target effects.^27^ These technologies are catalyzing a paradigm shift in genetic screening, endowing us with unprecedented versatility and accuracy. Moreover, within this section, we delve into a range of advanced techniques, encompassing multi-omics integration, *in vivo* and tissue-specific CRISPR screens, single-cell technology integration, high-throughput screening platform development, machine learning and data analysis tool integration, and the synergy of next-generation off-the-shelf cell therapies. These innovations collectively redefine the landscape of CRISPR-based screening methods.

### Integration of multi-omics approaches

Numerous state-of-the-art technologies have emerged as synergistic counterparts to CRISPR screens, facilitating the identification of novel tumor targets and augmenting the effectiveness of immunotherapies (Figure 4). Recent advancements in technology, particularly the emergence of CRISPR screen, have revolutionized the analysis of biological systems at multiple levels. These levels encompass various aspects such as DNA sequence data (genomics), RNA expression levels (transcriptomics), expression regulation levels (epigenomics), protein interaction levels (proteomics), and metabolite levels (metabolomics).^165^ Integrating these diverse biological datasets through multi-omics studies allows a comprehensive understanding of complex diseases such as cancer from a holistic perspective. Unlike investigations focused on a single omics level, multi-omics studies leverage information across different biological activities, providing unparalleled insights into disease mechanisms. To further enhance the efficiency and integration of CRISPR screen with other subfields of biological research, future endeavors aim to establish a unified framework connecting various tools and methodologies for data collection.^166^

### *In vivo* and tissue-specific CRISPR screens

Traditionally, CRISPR screens are carried out in an *ex vivo* environment. The integration of CRISPR editing into *in vivo* studies of cancer immunology can generate a more faithful model of cancer by tracking the interactions between tumor and immune cells in a tissue microenvironment.^41^ Loss-of-function and gain-of-function CRISPR screens have been implemented in somatic cells, with CRISPRa and CRISPRi used together.^167^ These applications provide the foundation for establishing patient-specific models, leading to precise medicine in the future. Notably, recent studies by Bahrami et al.^168^^,^^169^ and Wirth et al.^168^^,^^169^ have demonstrated the feasibility and potency of *in vivo* CRISPR-Cas9 functional screening in models of AML and chemoresistance, unveiling crucial genes such as *BCL2*, *BRIP1*, and *COPS2* that govern therapeutic outcomes.

Two models exist for *in vivo* CRISPR screens, indirect *in vivo* (transplant-based) and direct *in vivo* (autochthonous) screens. Indirect *in vivo* screen includes a transplantation of the mutagenized cell pool into a recipient animal, often subcutaneously, while direct *in vivo* screening requires delivery of CRISPR components to the target organ site through two delivery systems: viral vectors (e.g., AAVs, lentiviruses, and tissue-tropic viruses) and non-viral systems (e.g., nanoparticles, microinjection, electroporation).^167^^,^^170^ Autochthonous screening, while providing a more authentic representation of physiological contexts by targeting specific organs, faces challenges due to lower delivery and infection efficiency. Consequently, current direct *in vivo* screening is constrained by limitations in the scale of sgRNA library sizes that can be practically used.

### Integration of single-cell technologies

The combination of CRISPR screens with single-cell sequencing technologies, commonly known as CRISP-seq or Perturb-seq, offers a powerful approach for assessing gene function at the single-cell level.^154^^,^^155^^,^^160^ This method enables simultaneous characterization of gene perturbations and transcriptome profiling.^155^ With regard to immunotherapy, this technique allows precise identification of specific gene targets and gene signatures associated with anti-tumor or pro-tumor effects in tumor cells or effector cells, revealing fluctuations in cellular states in response to individual gene perturbations and corresponding genetic interactions.^154^^,^^155^^,^^160^ Ultimately, this integrative approach provides valuable insights into cellular heterogeneity, facilitates identification of rare cell populations, and elucidates cell-specific gene regulatory networks. identify rare cell populations, and elucidate cell-specific gene regulatory networks.^155^

### Development of high-throughput screening platform

Advancements in automation and miniaturization of CRISPR screens will enable higher throughput screening capabilities, which can be achieved by optimized gRNA libraries, improved delivery systems for gRNA and enzymes, advanced computational tools for screening result analysis, and updated functional assays for expanded gene candidate exploration.^171^^,^^172^^,^^173^ For instance, gRNA libraries have been refined to target coding and regulatory regions of drosophila, mouse, and human genomes with exceptional precision, while minimizing off-target effects.^171^^,^^174^ Multiple techniques including lentivirus transduction and lipid nanoparticle-loaded CRISPR complexes offer diverse options for gRNA delivery approaches.^172^ The enhanced high-throughput screening capacity, therefore, facilitates the investigation of larger gene sets, drug libraries, and complex genetic interactions, propelling the field of CRISPR-based research.

### Integration of machine learning and data analysis tools

Machine learning algorithms and advanced data analysis techniques are vital for extracting meaningful information from large-scale CRISPR screen datasets. Integrating computational approaches enhances interpretation, prediction, and prioritization of gene function and therapeutic targets. Currently, using machine deep learning (MDL)-based CRISPR techniques, alongside tools like DESeq2, edgeR, and CERES (Table 4),^175^^,^^176^ enables advanced feature selection, accurate classification and prediction of on-target activity, and interpretable visualization of screening results. These advancements drive progress in understanding gene function and potential therapeutics.

**Table 4:** Overview of technologies mentioned

Technologies	Acronym	Description	Advantages
CRISPR screen	CRISPR screen	use of gRNAs to direct the Cas9 enzyme to specific genomic sites, causing double-stranded breaks that trigger DNA repair processes, leading to either random mutations that disrupt gene function or the replacement of the target gene with foreign DNA sequences	aiding in assessing the functional consequences of individual gene knockouts or knockins in a high-throughput manner
Profiling relative inhibition simultaneously in mixtures	PRISM	pooled screening for a mixture of barcoded cell lines	enabling highly scalable screens
sgRNA lentiviral infection with Cas9 protein electroporation	SLICE	a hybrid system involving the introduction of traceable sgRNA cassettes using lentivirus and the delivery of Cas9 protein via electroporation	facilitating efficient and specific disruption of target genes, enhancing the monitoring and recognition of cells where gene disruptions have happened, and enlarging the scope of applicable primary cell types for screens
Single-cell RNA sequencing	scRNA-seq	analysis of nucleic acid sequence data of individual cells	elucidating cellular heterogeneity with a higher resolution
Cytometry by time of flight	CyTOF	quantification of the presence of metal isotope labels on antibodies and other markers at the single-cell level through mass spectrometry	preventing the interference caused by autofluorescence and spectral overlap
Perturbation sequencing/CRISPR-pooled screens sequencing/CRISPR droplet sequencing	Perturb-seq/CRISP-seq/CROP-seq	combinations of CRISPR screens with single-cell sequencing technologies	supporting large-scale gene perturbation studies, and allowing the evaluation of gene functions at the single-cell level
differential gene expression analysis on the basis of the negative binomial distribution	DESeq2	an R/Bioconductor software package used for analyses of comparative RNA-seq data by using shrinkage estimators for dispersion and fold change	improving the sensitivity and precision of screen results, leading to the emphasis on the strength of differential expression
Empirical analysis of digital gene expression (DGE) in R	edgeR	an R/Bioconductor software package used for evaluating variations in gene expression on the basis of replicated count data	enhancing the reliability of inference, and allowing analyses at the most minimal levels of replication
CERES	CERES	a computational approach for examining gene-dependency levels with CRISPR-Cas9 essentiality screens while considering the copy number-specific impact	reducing false-positive rates of screens

gRNA, guide RNA; sgRNA, single-guide RNA.

### Combination of next-generation off-the-shelf cell therapy

CRISPR screen techniques have emerged as a valuable tool for identifying and studying crucial genes involved in cancer immunotherapy.^27^ This knowledge can be applied to engineer advanced off-the-shelf cell therapies with improved functionality. Strategies such as eliminating HLAs and TCRs while incorporating the overexpression of inhibitory ligands like NK inhibitory ligands HLA-E, HLA-G, and macrophage inhibitory ligand CD47 have been shown to mitigate the adverse effects of graft-versus-host disease (GvHD) and host-versus-graft (HvG) responses in allogeneic cell products, thereby enhancing their safety.^177^^,^^178^^,^^179^^,^^180^ Precise CRISPR-mediated modifications in allogeneic cells allow for the disruption of immune checkpoints, manipulation of chemokine pathways, enhancement of T cell signaling, recruitment of anti-tumor immune cells, and modulation of the immunosuppressive TME.^73^^,^^181^^,^^182^^,^^183^ Additionally, CRISPR techniques hold great potential for manipulating and reprogramming cell fate, including hematopoietic stem cells (HSCs) and induced pluripotent stem cells (iPSCs), with the aim of developing stem-cell-derived therapeutic cell products for cancer immunotherapy.^184^^,^^185^^,^^186^^,^^187^^,^^188^^,^^189^^,^^190^

## Conclusion

Immunotherapy has emerged as an advanced approach in cancer treatment, demonstrating remarkable efficacy in cancer patients through the use of next-generation therapeutics such as CAR-T cell therapy, TIL therapy, checkpoint inhibitors, bispecific antibodies, oncolytic viral therapies, cancer vaccines, and others.^191^^,^^192^^,^^193^^,^^194^^,^^195^ Despite the significant progress made, there are certain limitations that hinder the achievement of maximum efficacy in cancer immunotherapy. These limitations encompass factors such as tumor heterogeneity, lack of tumor-specific antigens, tumor antigen escape, immune suppression, limited response in certain cancer types, immune-related adverse events, and other challenges.^196^^,^^197^^,^^198^ Consequently, there is an urgent need to develop novel technologies that can effectively overcome these existing limitations and swiftly address the prevailing difficulties encountered in cancer immunotherapy. Such advancements have the potential to transform our understanding of tumor immunology at a profound level and reshape the current landscape of cancer treatment paradigms.^179^^,^^199^

CRISPR screen technology has emerged as a pivotal tool in understanding the genetic basis of diseases, including cancer, and has revolutionized the field of cancer immunotherapy. This review offers a comprehensive overview of the applications and impact of CRISPR screen technology within the realm of cancer immunotherapy. By enabling systematic exploration of gene function, CRISPR screening has brought about a paradigm shift in functional genomics research. Leveraging the precision and adaptability of the CRISPR-Cas9 system, researchers can delve into the intricate complexities of gene networks and gain novel insights into biological processes and disease mechanisms. The continued refinement and application of CRISPR screening methodologies hold immense promise in unraveling new discoveries in genetics and propelling innovative therapeutic interventions to the forefront.
